# Supplementary figures and images for: Unravelling genetic differentiation between Glossina brevipalpis populations from two distant National Parks in Mozambique
Source: PLoS Negl Trop Dis. 2025 May 30;19(5):e0012953. doi: 10.1371/journal.pntd.0012953 (PMC12157922; doi:10.1371/journal.pntd.0012953)

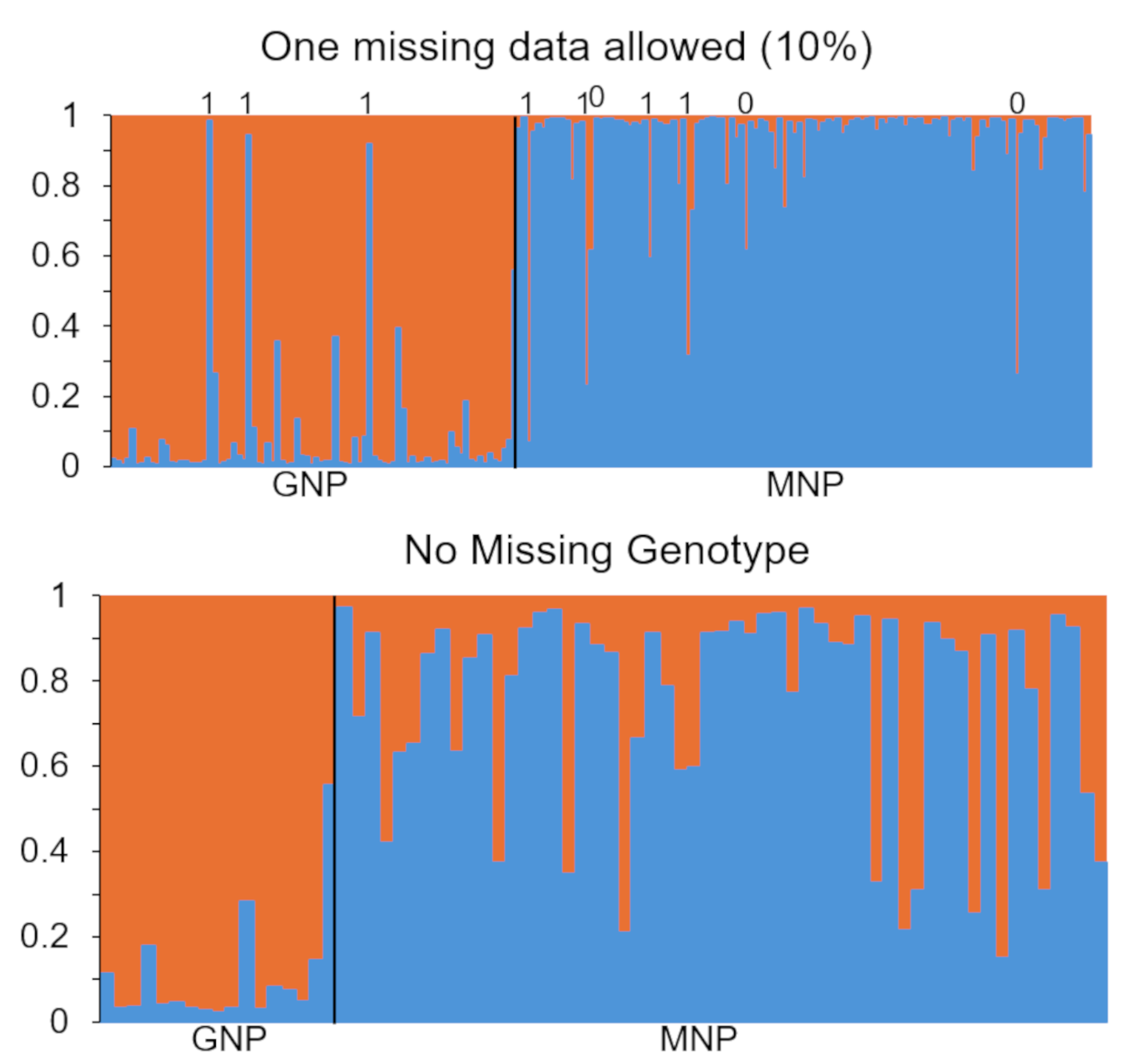

Supplement: S1 Fig — Numbers on the top indicate the number of missing genotypes for individuals assigned to their park of origin with less than 50% probability. Average probabilities of assignment to the park of origin was 0.9234 and 0.7811 for One missing and No missing datasets, respectively. (TIF) [file pntd.0012953.s004.tif]

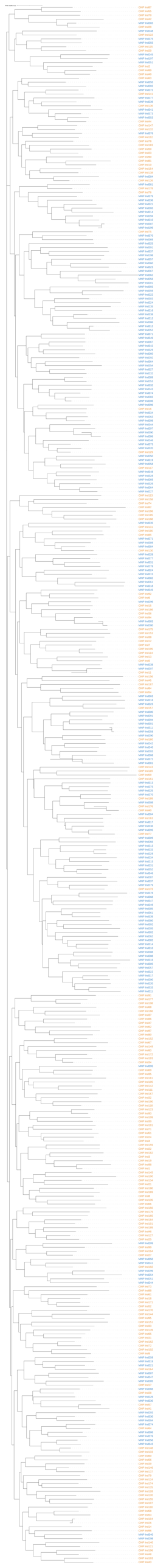

Supplement: S2 Fig — The tree is based on Cavalli-Sforza and Edward’s chord distance corrected for null alleles, built with MEGA X and edited with iTOL V6. (PDF) [file pntd.0012953.s005.pdf]

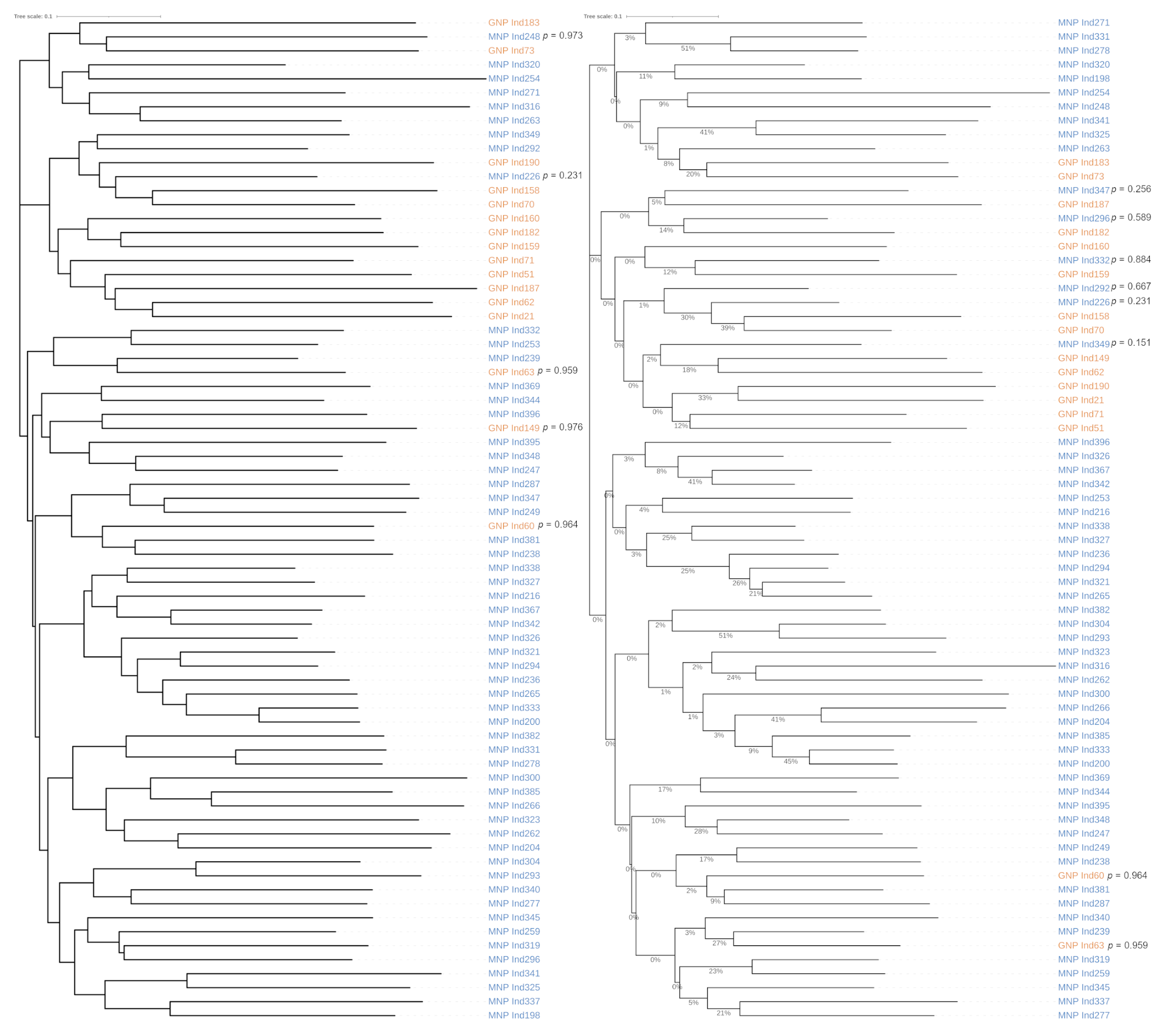

Supplement: S3 Fig — These trees are based on Cavalli-Sforza and Edward’s chord distance corrected for null alleles (left), or on Nei’s DA genetic distance (right). These were built with MEGA X and PopTree, respectively, and edited in iTOL V6. Probabilities in black are the assignment of corresponding individuals to their park of origin. (TIF) [file pntd.0012953.s006.tif]
