# Supplementary material for: Unravelling genetic differentiation between Glossina brevipalpis populations from two distant National Parks in Mozambique
Source: PLoS Negl Trop Dis. 2025 May 30;19(5):e0012953. doi: 10.1371/journal.pntd.0012953 (PMC12157922; doi:10.1371/journal.pntd.0012953)
Supplement: Attachment — Submitted filename: review.docx [file pntd.0012953.s007.docx]

This manuscript entitled « *Unravelling genetic differentiation between Glossina brevipalpis populations from two distant National Parks in Mozambique* » deals with the population genetics of a *Glossina brevipalpis*, a vector of Trypanosoma parasites, in Mozambique. The authors sampled and genotyped about 400 tsetse flies in two National Parks in Mozambique with 10 microsatellite markers. Tsetse distribution in Mozambique is discountinued with a southern fly belt (where Maputo National Park is) and a central belt (where Gorongosa National Park is). The main objective of this study is therefore to understand gene flow between the two parks.

The authors acknowledge that they « faced many genotyping problems with a substantial number of missing genotypes at several loci ». Nevertheless, they draw convincing conclusion on the high genetic differentiation between the two parks, with low migration rates (2 individuals per year). They discuss the hypothesis of pocket populations between the two parks or human-driven transportation of tsetse flies.

I think that the results section is a bit hard to follow, very technical while the introduction and/or discussion miss a bit of comparisons with what has already been published on tsetse flies population genetics.

Overall, I believe that this work brings useful information on the dispersal of tsetse flies, which could help designing more efficient control strategies.

My main concern is about the quality of the genotype data. Quality testing is presented after analyses in the Mat & Meth and mixed with analyses in the Results. It both complicates reading and bring doubts on the obtained results. I believe that the authors did the right tests and analyses but the presentation is not so clear. Also this paper lacks a complete discussion about published results on tsetse flies population genetics (to compare with the present results) and about the consequences of the results on vector control.

Major issues :

L52- 69 : This paragraph jumps from G. brevipalpis to the three other species, from Mozambique to Southern Africa, from past to present situation. Could you reorganize it so that it is more straighforward to read ?

L71-72 : Could you explain why « understanding the dynamics of the existing populations of Glossina is important » ? It seems that the previous control campaigns (without any knowledge on populations genetics) were quite efficient. What would be the added value of population genetic results for tsetse control ?

L74-75 : Please expand a bit on the important results obtained with population genetics on tsetse flies (here or in the discussion section).

L78 : Assessing genetic diversity is one goal of this paper, but you don’t clearly present and discuss your results regarding genetic diversity (it is not even mentioned in the abstract). Is one park more genetically diverse than the other one ?

L107 : Supplementary Table S1 is not easy to read. Could you please provide a summary table with the locations GPS points, number of traps, number of individuals trapped and genotyped (males and females) ?

In the results section, could you provide a table with the main statistics (Sample size, number of alleles, observed and expected heterozygosity, Fis) ? A figure with Fis for each locus would also help (in supplementary maybe).

L325 : It seems that only individuals with missing genotypes are miss-assigned in the STRUCTURE analysis. Therefore I would expect you to rerun all the analyses (not only STRUCTURE) with a dataset excluding individuals with high levels of missing genotypes. Did you do that ? What is the impact on the results ?

In the discussion section, I expected that you compare your results with previously published ones on tsetse flies. Are they comparable ? Different ? Why ?

L396 : It is not clear to me what recommendations emerge from your study. I guess that the main message is that there is a risk of « reinvasions over very long distance ». Could you expand a bit on that ?

Minor issues :

L 22 : « tsetse- free » there is an extra-space after –

In the introduction : why did you choose to study G. brevipalpis instead of G. austeni (they seem to have the same distribution) ?

L55 : To which Glossina group do G. austeni, G. morsitans and G. pallidipes belong ?

L104-105 : Fig1 : I find it a bit difficult to compare the sampling scheme between the two sites since only a portion of Gorongosa National Park is represented. Also, in Maputo National Park, we can see several points in some locations (M02, M01). I supposed that this is because they are further apart than in other locations but you stated that in each location, the traps covered a total distance of 1 km. Could you clarify this ?

L129 : Did you analyse your dataset taking into account the sex of flies to analyse sex-biased dispersal ?

L146 : There is an extra space after (1994)

L157 : I would suggest to present quality testing of the data before data analysis.

In the results section, did you test isolation by distance (inside the two parks ?)

L233 : It is not clear to me if this dataset remained the same after the exclusion of 2 loci (over 10)

L237 : I don’t understand your sentence. Is there an extra « and » before « between All… » ?

L276 : Do you mean BLAST instead of « BLAT » ?

L295 : I think it can be useful to remind the reader what Hex and CoA stand for here. For example you can write « two in MNP for Hex and CoA methods » or give the full explanation « Two in MNP for heterozygote excess and Coancesty methods ».

L298 : I’m not used to read « minimax ». Is it a way to present of range of values ?

L303 : « with a minimum and maximum minimax = ». This sounds a bit strange (redundant) to me.

L308 : « There was no obvious signature of a bottleneck ». Is it because the 2 models don’t agree (one being significant and the other not) ?

L311-322: Do you use the dataset with the 10 markers ? or only 8- ? When mentioning 25%, 30% or 40% of missing data (or 90% genomic match), is it considering 10 or 8 markers ?

L358 : How do you explain that there is no signature of bottleneck despite the rinderpest pandemic and the vector control campaign ?
