# Supplementary material for: Unravelling genetic differentiation between Glossina brevipalpis populations from two distant National Parks in Mozambique
Source: PLoS Negl Trop Dis. 2025 May 30;19(5):e0012953. doi: 10.1371/journal.pntd.0012953 (PMC12157922; doi:10.1371/journal.pntd.0012953)
Supplement: Attachment — Submitted filename: Response to Reviewers.docx [file pntd.0012953.s009.docx]

Rebuttal letter

Dear Editor,

We have now reshaped the manuscript and made additional analyses, which were requested by the referees, and which are now reported in the new manuscript (see below).

Please also note that we have added supplementary effective population size estimates that explain the very small differences with previous results.

Please find below the answers to all referees' and editors’ queries.

Reviewer #1: The conclusions are generally supported by the data presented, with the observed genetic differentiation between populations and evidence of rare immigration between parks being consistent with the findings. However, some of the conclusions, such as the impact of motorized transport on tsetse dispersal, would benefit from further clarification and supporting evidence.

The limitations of the analysis are acknowledged, particularly in relation to the challenges posed by missing genotypes and the potential bias in the Bayesian clustering process.

*Answer: We thank referee 1 for her comments. We have added some new analyses (supplementary figures) and additional comments in the discussion section. We hope this will meet Referee1's satisfaction.*

Reviewer #2: In the discussion section, I expected that you compare your results with previously published ones on tsetse flies. Are they comparable ? Different ? Why ?

*Answer: We have now added some comments in our discussion regarding other species. Nevertheless, G. brevipalpis belongs to the Fusca group of tsetse flies, which has been poorly studied so far, so that comparisons are somehow difficult.*

It is not clear to me what recommendations emerge from your study. I guess that the main message is that there is a risk of « reinvasions over very long distance ». Could you

expand a bit on that?

*Answer: We have added some additional recommendations in our conclusion. We hope it will meet Referee2's satisfaction.*

Editorial and Data Presentation Modifications?

Use this section for editorial suggestions as well as relatively minor modifications of existing data that would enhance clarity. If the only modifications needed are minor and/or editorial, you may wish to recommend “Minor Revision” or “Accept”.

Reviewer #1: Minor revision

Reviewer #2: (No Response)

Summary and General Comments

Use this section to provide overall comments, discuss strengths/weaknesses of the study, novelty, significance, general execution and scholarship. You may also include additional comments for the author, including concerns about dual publication, research ethics, or publication ethics. If requesting major revision, please articulate the new experiments that are needed.

Reviewer #1: The paper presents an important study on the genetic differentiation of *Glossina brevipalpis* populations in Mozambique using microsatellite markers. Understanding gene flow and population structure is essential for effective vector control strategies. The paper is very solid though the findings are entirely expected given the spatial scale of the sampling and the existence of obvious variance (deduced from the authors own admissions). One thing that I have to give it up to the authors is the degree of thoroughness and robustness in the statistical approaches; the analyses were well handled. I have few comments and/or specific questions.

1. The abstract can and should conclude with a stronger statement on the implications for vector control (if any).

*Answer: We have added a sentence of recommendations at the end of the abstract*

2. Line 28: The last sentence of the abstract offers a great implication for further research, but might benefit from slight rewording for clarity: “We suggest that undocumented means of dispersal, such as translocation through human-driven animal movement, should be explored in future studies investigating Glossina populations”

*Answer: done*

3. The human-driven transport hypothesis is plausible but not directly tested. The authors should either provide supporting evidence (e.g., known livestock transport routes) or acknowledge this as a limitation.

*Answer: We respectfully advise Referee1 that we already mentioned documented animal transport routes in the discussion (Line 395 - 399). As far as domestic animals are concerned, there is evidence of limited livestock transportation (according to the Ministry of Agriculture, Environment and Fisheries) between the regions where the two parks are located, which might have allowed for the migration of some G. brevipalpis specimens. We have added this information to the discussion.*

4. Line 100-101: What was the average distance between the traps in each location?

*Answer: This has been added in the text in the material and method section*

5. Make sure that each figure has a detailed caption that helps the reader understand what is being presented. For example, in the Figure 1; state clearly what the G1-G4 and M1-M4) represent; it’s not very obvious that it relates to the Park names!

*Answer: done*

6. Line 110. 402 samples were selected for genotyping based on the location and condition of specimen. What happened to the rest of the samples??

*Answer: We decided to use a minimum of 200 samples per parks since it is representative of the populations. The remaining samples were used for another study (i.e. blood meal identification with metabarcoding). We now mention this in the Material and Methods section.*

7. Line 180: Is it “recoded” or “recorded”? This has been repeated severally if it’s not “recoding”

*Answer: done The term is correct since what we are saying is that the missing data were recoded for the analysis with FreeNA*

8. Line 96. According to the authors’ own descriptions, it appears several species of *Glossina* occur sympatrically in the sampled areas. How did you definitively identify *brevipalpis* for genotyping? This is not described in the methods.

*Answer: We have added how G. brevipalpis was identified in the Material and Methods section.*

9. Line 96-98: Just out of curiosity, why did you use two different trap types (NGU and H-traps) for sampling?

*Answer: This is because we were not just sampling for this particular study. We also wanted to capture other species of tsetse flies for another study on species diversity. While H-traps are best for capturing G. brevipalpis, some were caught in NGUs or by a hand net (in Maputo National Park), which is why we mentioned them. We now specify this in the Material and Methods section.*

10. Lines 343-346: How might these mis-assignments affect the inferences about migration and gene flow?

*Answer: As explained in the text, gene flow or dispersal were estimated from F_ST_ corrected for null alleles and polymorphism excess, and we know that missing data are ignored by the algorithms used to undertake these estimates. We thus do not expect strong biases in this respect. For STRUCTURE, to our knowledge, there is no way to account for null alleles, and our results show that missing data are obviously used in the assignment algorithm, leading to fake genetic distances between individuals that are the most affected and between those and the other individuals. We have added some comments on this issue.*

11. Lines 390-392. Again on the suggestion of motorized human transport as being responsible for the exchange of individuals between the parks, this is an interesting hypothesis that warrants further exploration. Could the authors provide a more detailed discussion of how such transport could occur and whether there are documented instances of tsetse flies being accidentally transported by humans, vehicles, or animals?

*Answer: We think we have already answered this point above*

Reviewer #2: This manuscript deals with the population genetics of a *Glossina brevipalpis*, a vector of *Trypanosoma* parasites, in Mozambique. The authors sampled and genotyped about 400 tsetse flies in two National Parks in Mozambique with 10 microsatellite markers. Tsetse distribution in Mozambique is discountinued with a southern fly belt (where Maputo National Park is) and a central belt (where Gorongosa National Park is). The main objective of this study is therefore to understand gene flow between the two parks.

The authors acknowledge that they « faced many genotyping problems with a substantial number of missing genotypes at several loci ». Nevertheless, they draw convincing conclusion on the high genetic differentiation between the two parks, with low migration rates (2 individuals per year). They discuss the hypothesis of pocket populations between the two parks or human-driven transportation of tsetse flies.

I think that the results section is a bit hard to follow, very technical while the introduction and/or discussion miss a bit of comparisons with what has already been published on tsetse flies population genetics.

*Answer: We think we have already answered that point above.*

Overall, I believe that this work brings useful information on the dispersal of tsetse flies, which could help designing more efficient control strategies.

My main concern is about the quality of the genotype data. Quality testing is presented after analyses in the Mat & Meth and mixed with analyses in the Results. It both complicates reading and bring doubts on the obtained results. I believe that the authors did the right tests and analyses but the presentation is not so clear.

*Answer: We have amended the structure of the Material and Methods section to make it clearer, and hope this will meet Referee2's satisfaction.*

Also this paper lacks a complete discussion about published results on tsetse flies population genetics (to compare with the present results) and about the consequences of the results on vector control.

*Answer: G. brevipalpis has been poorly studied and only recently have microsatellites for this species become available. Nevertheless, we have added some information about other species.*

PLOS authors have the option to publish the peer review history of their article (what does this mean?). If published, this will include your full peer review and any attached files.

If you choose “no”, your identity will remain anonymous but your review may still be made public.

Do you want your identity to be public for this peer review? For information about this choice, including consent withdrawal, please see our Privacy Policy.

Reviewer #1: Yes: Robert Opiro

Reviewer #2: Yes: Hélène Jourdan-Pineau

Reviewer 3

This manuscript entitled « *Unravelling genetic differentiation between Glossina brevipalpis populations from two distant National Parks in Mozambique* » deals with the population genetics of a *Glossina brevipalpis*, a vector of Trypanosoma parasites, in Mozambique. The authors sampled and genotyped about 400 tsetse flies in two National Parks in Mozambique with 10 microsatellite markers. Tsetse distribution in Mozambique is discountinued with a southern fly belt (where Maputo National Park is) and a central belt (where Gorongosa National Park is). The main objective of this study is therefore to understand gene flow between the two parks.

The authors acknowledge that they « faced many genotyping problems with a substantial number of missing genotypes at several loci ». Nevertheless, they draw convincing conclusion on the high genetic differentiation between the two parks, with low migration rates (2 individuals per year). They discuss the hypothesis of pocket populations between the two parks or human-driven transportation of tsetse flies.

I think that the results section is a bit hard to follow, very technical while the introduction and/or discussion miss a bit of comparisons with what has already been published on tsetse flies population genetics.

Overall, I believe that this work brings useful information on the dispersal of tsetse flies, which could help designing more efficient control strategies.

My main concern is about the quality of the genotype data. Quality testing is presented after analyses in the Mat & Meth and mixed with analyses in the Results. It both complicates reading and bring doubts on the obtained results. I believe that the authors did the right tests and analyses but the presentation is not so clear. Also this paper lacks a complete discussion about published results on tsetse flies population genetics (to compare with the present results) and about the consequences of the results on vector control.

*Answer: We think that this part has been copied and pasted from Referee2's comments (see our answers above)*

Major issues:

L52- 69: This paragraph jumps from *G. brevipalpis* to the three other species, from Mozambique to Southern Africa, from past to present situation. Could you reorganize it so that it is more straightforward to read?

*Answer: done*

L71-72: Could you explain why « understanding the dynamics of the existing populations of *Glossina* is important » ? It seems that the previous control campaigns (without any knowledge on populations genetics) were quite efficient. What would be the added value of population genetic results for tsetse control ?

*Answer: done*

L74-75: Please expand a bit on the important results obtained with population genetics on tsetse flies (here or in the discussion section).

*Answer: We think we already answered that point above.*

L78: Assessing genetic diversity is one goal of this paper, but you don’t clearly present and discuss your results regarding genetic diversity (it is not even mentioned in the abstract). Is one park more genetically diverse than the other one ?

*Answer: We think we already answered that point above*

L107: Supplementary Table S1 is not easy to read. Could you please provide a summary table with the locations GPS points, number of traps, number of individuals trapped and genotyped (males and females) ?

In the results section, could you provide a table with the main statistics (Sample size, number of alleles, observed and expected heterozygosity, Fis) ? A figure with Fis for each locus would also help (in supplementary maybe).

*Answer: done*

L325: It seems that only individuals with missing genotypes are miss-assigned in the STRUCTURE analysis. Therefore I would expect you to rerun all the analyses (not only STRUCTURE) with a dataset excluding individuals with high levels of missing genotypes. Did you do that ? What is the impact on the results ?

*Answer: We have added results with only one or no missing data per individuals. Though probabilities of assignment are affected, this did not change the general picture. The existence of rare exchange of immigrants seems valid. This was confirmed by a NJTree analysis that we have added in our results.*

In the discussion section, I expected that you compare your results with previously published ones on tsetse flies. Are they comparable ? Different ? Why ?

*Answer: We have already answered this point above.*

L396: It is not clear to me what recommendations emerge from your study. I guess that the main message is that there is a risk of « reinvasions over very long distance ». Could you expand a bit on that ?

*Answer: We think we already answered that point above.*

Minor issues:

L 22: « tsetse- free » there is an extra-space after –

*Answer: done*

In the introduction: why did you choose to study G. brevipalpis instead of G. austeni (they seem to have the same distribution) ?

*Answer: They have a similar distribution, not necessarily the same. There are no available published microsatellites for G. austeni so far, and the overall project was focusing on two National Parks; and there was no recent evidence of G. austeni presence in Gorongosa National Park.*

L55: To which Glossina group do G. austeni, G. morsitans and G. pallidipes belong ?

*Answer: Morsitans*

L104-105: Fig1: I find it a bit difficult to compare the sampling scheme between the two sites since only a portion of Gorongosa National Park is represented. Also, in Maputo National Park, we can see several points in some locations (M02, M01). I supposed that this is because they are further apart than in other locations but you stated that in each location, the traps covered a total distance of 1 km. Could you clarify this ?

*Answer: Concerning Gorongosa National Park, to better visualize the four sampling sites, we zoomed in on them on the map. As described in the paper, we selected four sampling sites in Maputo as well, each focus site has 2 to 6 traps. There was also an error in the map, which has been corrected.*

L129: Did you analyse your dataset taking into account the sex of flies to analyse sex-biased dispersal?

*Answer: Yes, the gender was available. Nevertheless, given the almost absence of exchange between the two parks, there is no way we could detect a genetic signature of sex biased dispersal (see for instance the simulation study in Goudet et al (Goudet J, Perrin N, Waser P (2002) Tests for sex-biased dispersal using bi-parentally inherited genetic markers. Molecular Ecology, 11, 1103-1114.* [*https://doi.org/10.1046/j.1365-294X.2002.01496.x*](https://doi.org/10.1046/j.1365-294X.2002.01496.x)*). We nevertheless undertook it within each park separately, and found no signature of sex-biased dispersal. This was added in the manuscript.*

L146: There is an extra space after (1994)

*Answer: done*

L157: I would suggest to present quality testing of the data before data analysis.

*Answer: The first quality control test was to determine at what level population subdivision occurred, before testing for the quality of loci. We have rephrased the heads of paragraphs to make this clearer.*

In the results section, did you test isolation by distance (inside the two parks ?)

*Answer: Given the lack of subdivision within the parks and the reduced number of individual per site or trap within the parks we originally did not test for isolation by distance. To answer Referee 3's query, we undertook it separately, within each park, between traps. We expectedly found no significant genetic signature of isolation by distance, confirming that tsetse flies move freely, or almost so, across the entire investigated zone within each park. This result is now shortly discussed in the new manuscript.*

L233: It is not clear to me if this dataset remained the same after the exclusion of 2 loci (over 10)

*Answer: We are not sure what is the point raised by the referee here. The number of samples (individuals) remained the same. Only the number of loci decreased from 10 to 8 in our final analyses. We explained (hopefully well enough) why we needed to exclude these two loci in the results section.*

L237: I don’t understand your sentence. Is there an extra « and » before « between All… » ?

*Answer: What we mean is that significance was only detected when looking at F_IS_ and LD, and only when comparing All (no subdivsion) to the other 3 sampling designs.*

L276: Do you mean BLAST instead of « BLAT »?

*Answer: BLAT is a tool similar to BLAST optimized to align your query sequence to your target genome (Kent WJ. BLAT—The BLAST-Like Alignment Tool. Genome Res. 2002;12: 656–664. doi:10.1101/gr.229202)*

L295: I think it can be useful to remind the reader what Hex and CoA stand for here. For example you can write « two in MNP for Hex and CoA methods » or give the full explanation « Two in MNP for heterozygote excess and Coancesty methods ».

*Answer: done*

L298: I’m not used to read « minimax ». Is it a way to present of range of values?

*Answer: We corrected the typo.*

L303: « with a minimum and maximum minimax = ». This sounds a bit strange (redundant) to me.

*Answer: done*

L308: « There was no obvious signature of a bottleneck ». Is it because the 2 models don’t agree (one being significant and the other not)?

*Answer: As explained in the Material and Methods section, a genetic signature of a past bottleneck can only be assessed if at least IAM and TPM provide a significant p-value.*

L311-322: Do you use the dataset with the 10 markers? or only 8- ? When mentioning 25%, 30% or 40% of missing data (or 90% genomic match), is it considering 10 or 8 markers?

*Answer: All analyses were done using 8 markers as stated in the text (L301 - 288). For STRUCTURE and NJTree analyses, we also checked with individuals that displayed no more than 2 missing loci (25%), 1 missing locus (12.5%) and 0 missing data (complete genotypes at the 8 loci).*

L358: How do you explain that there is no signature of bottleneck despite the rinderpest pandemic and the vector control campaign?

*Answer: We have added an explanation in the discussion. Immigration events from other areas not included in our study may have brought enough exogenous alleles to mask any bottleneck signature.*

We hope that our article is now suitable for publication in PLoS NTD and remain at your disposal for any modification or questions you may find necessary.

Sincerely

Denise Brito

Thierry de Meeûs

Luis Neves
